# Successful excision of an inflammatory endobronchial polyp using biopsy forceps with improvement in FEV1 in a patient with allergic bronchopulmonary aspergillosis: A case report^☆^

**DOI:** 10.1016/j.rmcr.2024.102011

**Published:** 2024-03-11

**Authors:** Fumihiro Kashizaki, Kenji Konishi, Chihiro Yamada, Shunsuke Okazaki, Hao Chen, Atsushi Miyasaka, Nanami Tsuchiya, Akitomo Kikuchi, Kentaro Yumoto, Yui Kojima, Hiroyuki Osawa, Harumi Koizumi, Kenichi Takahashi, Takeshi Kaneko

**Affiliations:** a Department of Respiratory Medicine, Yokohama Minami Kyosai Hospital, Yokohama, Japan; b Department of Respiratory Medicine, Seirei Yokohama Hospital, Yokohama, Japan; c Department of General Thoracic Surgery, Yokohama Minami Kyosai Hospital, Yokohama, Japan; d Department of Pathology, Yokohama Minami Kyosai Hospital, Yokohama, Japan; e Department of Respiratory Medicine, Yokohama City University Hospital, Yokohama, Japan

**Keywords:** Allergic bronchopulmonary aspergillosis, Inflammatory endobronchial polyps, Biologic, Biopsy forceps, Flexible bronchoscopy, Pulmonary function test

## Abstract

Inflammatory endobronchial polyps (IEPs) are rare, benign bronchial tumors posing diagnostic and therapeutic challenges owing to limited data. A 55-year-old man, receiving treatment for allergic bronchopulmonary aspergillosis, presented with a one-week history of fever and purulent sputum. Diagnosed with pneumonia, he received antimicrobial treatment. However, because of persistent symptoms, an endobronchial tumor was suspected on computed tomography. IEP was confirmed through flexible bronchoscopy with forceps biopsy, and polyp removal improved symptoms, lung function, and imaging.

## Highlights

• IEPs are rare benign bronchial tumors caused by chronic respiratory diseases.
• The appropriate treatment of IEPs should be selected considering comorbidities.
• Monitoring lung function is crucial in IEPs patients with chronic lung diseases.
• Data on the recurrence of IEPs are scarce owning to the rarity of this disease.

## 1 Introduction

Inflammatory endobronchial polyps (IEPs) are rare, benign bronchial tumors caused by infection, trauma, or chronic respiratory diseases [[1], [2], [3]]. Owing to insufficient data on IEPs, the management of patients with IEPs with comorbid chronic respiratory diseases presents diagnostic and therapeutic challenges. Furthermore, the impact of IEP on lung function varies depending on its location. Preserved lung function is a key prognostic factor in patients with chronic lung diseases, such as bronchial asthma, chronic obstructive pulmonary disease, and interstitial lung disease. Therefore, monitoring posttreatment lung function is crucial in patients with chronic respiratory diseases. In addition, respiratory complications must be carefully considered during diagnosis, and treatment strategies that preserve lung function must be selected for tumor removal. To the best of our knowledge, this is the first case of IEP in a patient with allergic bronchopulmonary aspergillosis (ABPA) on biological therapy that confirmed FEV1 improvement after polyp removal using flexible bronchoscopic biopsy forceps.

## 2 Case presentation

**Fig. 1:**
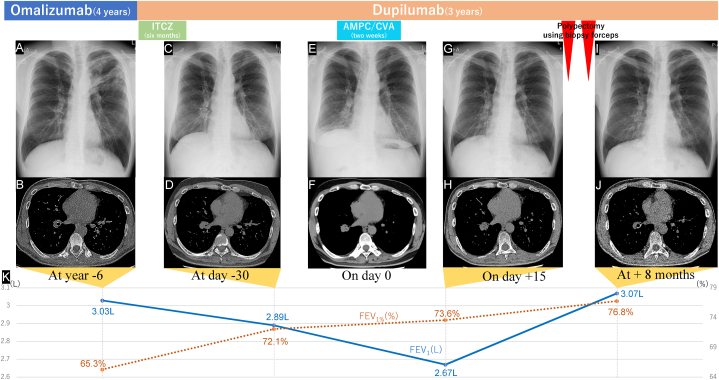
Clinical course on chest images and PFT (A) Chest radiograph at year −6 shows ill-defined consolidation, nodules, and opacities along the bronchus in the left upper lobe, accompanied by bronchial wall thickening. (B) Chest CT at year −6 shows no apparent intratracheal lesions in the right lower lung lobe. (C) Chest radiograph at day −30 shows significant improvement in the consolidation in the left upper lung lobe. However, irregular opacities around the truncus intermedius and elevation of the diaphragm on the right side are observed. (D) Chest CT at day −30 shows constriction of the lumen of the truncus basalis. (E) Chest radiograph on day 0 shows right basilar opacities with volume loss and further elevation of the diaphragm on the right side. (F) Chest CT on day 0 shows complete occlusion of the truncus basalis with a low-density area containing mucus at the center of the occluded lesion. (G) Chest radiograph on day +15 shows improved right basilar opacities and a partial reduction in the diaphragm elevation on the right side. (H) Chest CT on day +15 shows an endobronchial soft tissue nodule in the truncus basalis without a low-density area. (I) Chest radiograph at + 8 months shows no new lesions. Although linear and reticular shadows persist, the right lower lobe bronchus is preserved. (J) Chest CT at + 8 months shows complete resolution of the obstruction in the truncus basalis of the right bronchial lumen. (K) PFTs conducted two months after omalizumab initiation reveal an FEV1% of 65.3%, with an FEV1 of 3.03 L. On day -30, PFTs reveal an FEV1% of 72.1% and an FEV1 of 2.89 (L) On day +15, FEV1% and FEV1 are 73.6% and 2.67 L, respectively. At +8 months, the FEV1% and FEV1 values have improved to 76.8% and 3.07 L, respectively. Abbreviations: CT, computed tomography; PFT, pulmonary function test; ITCZ, itraconazole; AMPC/CVA, amoxicillin-clavulanate; FEV1%, forced expiratory volume in one second as a percent of forced vital capacity; FEV1, forced expiratory volume in one second.

**Fig. 2:**
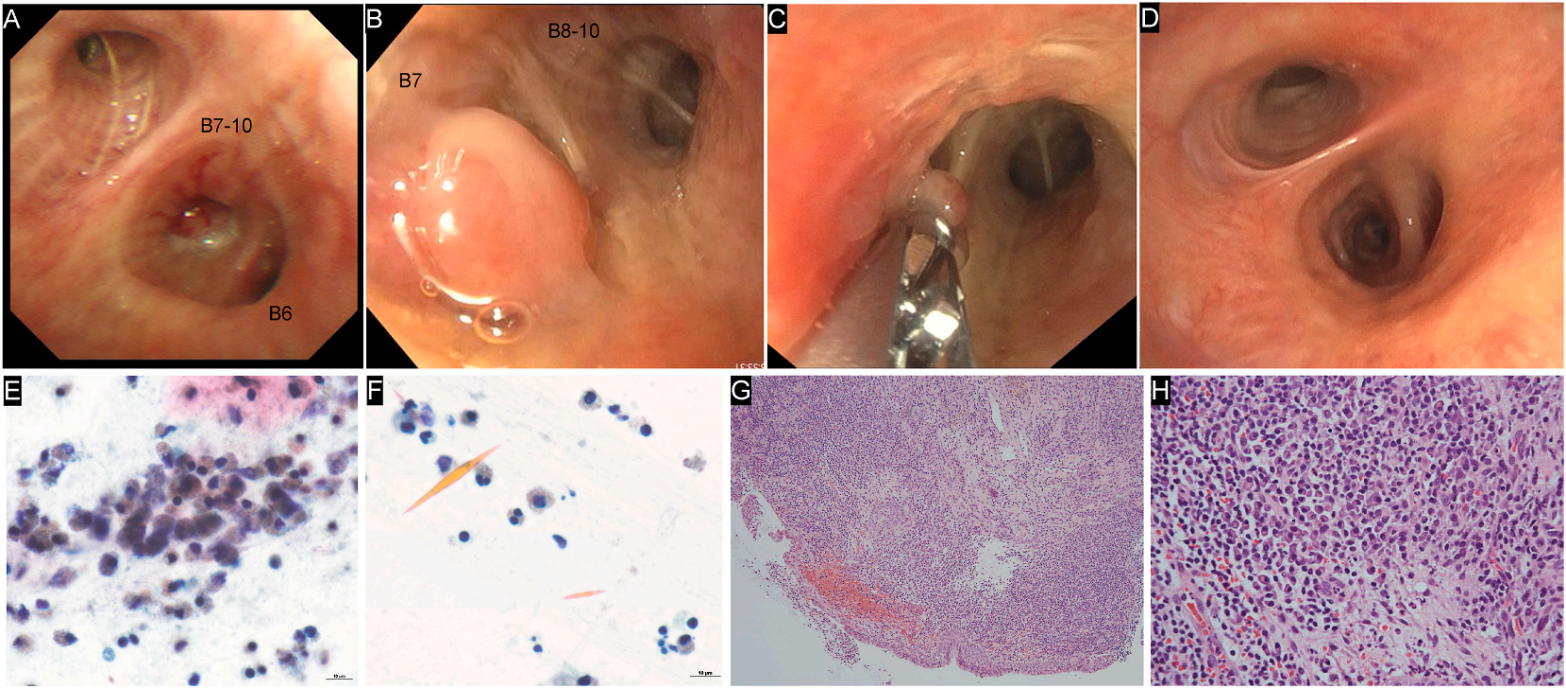
Bronchoscopic and histopathological findings (A) Bronchoscopic image (day +45) showing almost complete occlusion of the right truncus basalis by a smooth nodule surrounded by purulent mucus. *Aspergillus fumigatus* was cultured from the purulent mucus. (B) Two weeks after EBFB (day +60), a smooth pedunculated nodule obstructing the right B7 persists. (C) The nodule at the right B7 is removed using biopsy forceps. The bronchial mucosa on the mediastinal side of the biopsy forceps shows edema and redness owing to the previous nodule removal. (D) Bronchoscopic findings from the same angle as (A) at +8 months. The bronchial lumen shows improvement in edema of the bronchial mucosa due to nodule removal and no new lesions. (E), (F) Sputum cytology six years prior to flexible bronchoscopic intervention shows Charcot–Leyden crystals (Giemsa staining; F) with numerous eosinophils in the mucus (E). (G) and (H) Histopathological examination of the nodule shows the proliferation of inflammatory cells, predominantly lymphocytes and plasma cells, along with few fibroblasts. No atypical cells are observed. (G) Low- and (H) high-magnification images (hematoxylin and eosin stain). Abbreviations: EBFB, endobronchial forceps biopsy.

A 55-year-old Japanese man, a former smoker with a 20 pack-year history, who was under our care for ABPA, presented with fever, purulent sputum, right-sided chest pain, and cough lasting one week (day 0). His medical history included bronchial asthma and allergic rhinitis. The treatment for bronchial asthma included inhaled corticosteroids, long-acting β2 agonists, and long-acting muscarinic antagonists. The patient was diagnosed with ABPA based on the following findings: high attenuation mucus in the left upper lobe bronchus on chest computed tomography (CT); an *Aspergillus fumigatus*-specific IgE level of 5.30 kUA/L, a serum total IgE level of 802 kU/L, a white blood cell count of 6910/μL, and an eosinophil count of 988/μL on hematological examination; Charcot–Leyden crystals with numerous eosinophils in the mucus on sputum cytology; and a positive sputum culture for *Aspergillus fumigatus*. After the diagnosis of ABPA was confirmed, the patient initially received omalizumab (600 mg subcutaneously every other week) because prednisolone caused insomnia (Fig. 1, Fig. 2). However, because of poorly controlled nasal obstruction and ABPA, he was switched to dupilumab (600 mg subcutaneously initially followed by 300 mg subcutaneously every other week), and itraconazole (200 mg every 24 h) was added for six months. The symptoms were well-controlled until the patient's recent visit. Chest CT on day −30 revealed a collapsed lumen of the truncus basalis with slight atelectasis (Fig. 1D); therefore, further examination was planned.

On day 0, the patient's vital signs were temperature, 37.4 °C and SpO_2_, 95% (room air), and physical examination revealed rhonchi and decreased breath sounds in the right lower lung lobe. Hematological examination revealed a white blood cell count of 11090/μL (neutrophils 79.8%) and C-reactive protein (CRP) level of 9.28 mg/dL. All sputum cultures were negative. Chest radiography revealed right basilar consolidation, opacities, and diaphragmatic elevation (Fig. 1E). Chest CT revealed complete occlusion of the truncus basalis, with a central low-density area containing mucus (Fig. 1F). Based on an initial diagnosis of obstructive pneumonia, amoxicillin-clavulanate was administered for two weeks. However, cough and purulent sputum persisted, and repeat blood tests (Table 1) on day +15 revealed a slight elevation in CRP levels. Additionally, chest radiography showed persistence of right basilar opacities with slight volume loss, linear shadows, reticular shadows, and right-sided diaphragmatic elevation (Fig. 1G). Furthermore, chest CT revealed an endobronchial soft-tissue nodule in the truncus basalis with peripheral atelectasis (Fig. 1H).

**Table 1:** Baseline characteristics of the case.

Hematological data			Biochemical data		
White blood cell count	6010	/μL	Total protein	7.3	g/dL
Neutrophils	56.1	%	Albumin	4.2	g/dL
Lymphocytes	37.4	%	Aspartate aminotransferase	21	U/L
Monocytes	3.5	%	Alanine aminotransferase	21	U/L
Eosinophils	2.5	%	Gamma-Glutamyl transpeptidase	22	U/L
Basophils	0.5	%	Alkaline Phosphatase	69	U/L
Hemoglobin	14.8	g/dL	Lactate Dehydrogenase	141	U/L
Platelet	276	10^3^/μL	Creatinine	0.98	mg/dL
			Blood urea nitrogen	14.7	mg/dL
**Serological data**			Sodium	141	mmol/L
C-reactive protein	0.24	mg/dL	Potassium	4.3	mmol/L
Carcinoembryonic Antigen	2.0	ng/mL	Chlorine	104	mmol/L
IgE	16.1	kU/L	Calcium	8.6	mg/dL
Aspergillus-Ag	0.2				

Flexible bronchoscopy on day +45 (Fig. 1G and H) revealed an obstructing smooth nodule in the truncus basalis, surrounded by purulent mucus (Fig. 2A). A nodal biopsy was performed using endobronchial forceps with minimal bleeding and no other complications. The culture of the purulent mucus was positive for *Aspergillus fumigatus*. Histopathological findings revealed irregular interstitial fibrosis, hypervascularization with small blood vessels, and dense inflammatory-cell infiltrates, primarily comprising lymphocytes, plasma cells, and neutrophils. The IgG4-/IgG-positive cell ratio was <15%. No atypical cells were observed, consistent with inflammatory granulomas (Fig. 2G and H). Owing to the residual symptoms, a second bronchoscopic procedure was performed on day +60, which revealed a residual smooth pedunculated nodule obstructing the right B7 (Fig. 2B). An endobronchial forceps biopsy (EBFB) was performed (Fig. 2C), and the histopathological findings were consistent with the previous results, confirming the diagnosis of IEP. During all endoscopic procedures, argon plasma coagulation and cryobiopsy devices were available for managing bleeding and tumor removal, respectively. However, they were not required as EBFB proved sufficient. All test results at +8 months showed improvement (Fig. 1, Fig. 2D).

## 3 Discussion

Approximately 5–10% of all benign bronchial tumors can be diagnosed [3]. IEPs are benign bronchial tumors first described as bronchial inflammatory polyps by Yankauer in 1929 [4]. Histopathologically, IEPs are inflammatory granulation tissues with varying proportions of inflammatory cells, fibroblasts, microvascular dilation, and fibrosis.

The pathogenesis of IEPs is thought to be damage to the airway mucosa caused by a trigger, leading to increased vascular permeability, edema, inflammatory cell infiltration, and fibrosis, causing mucosal herniation into the bronchial lumen [2,5]. Infectious diseases such as influenza and tuberculosis, allergies affecting the airway mucosa, foreign bodies, smoking, trauma, surgical invasions, and respiratory conditions such as bronchial asthma, cystic fibrosis, and silicosis have been reported as triggers for polyp formation [1,2,6,7]. While detailed reports on ABPA with concurrent IEPs are lacking, the pathology obtained suggests that these two diseases may have developed independently; however, several factors contribute to their development. One contributing factor is the robust and sustained immune response triggered by organisms like *Aspergillus* decomposing in the airways [8]. This response results in mucosal edema, increased vascular permeability, and inflammatory cell infiltration, all of which impair airway drainage. The presence of highly viscous mucus plugs further weakens the mucosa, making it more prone to damage. Roberts et al. reported that stagnation of purulent secretions plays a significant role in polyp formation [2]. Furthermore, coughing during mucus plug expulsion may exert a force that projects the mucosa into the bronchial lumen. Although the exact etiology of IEP in our patient remains unknown, possible factors include the duration of itraconazole administration, choice of biologic, use of corticosteroids, and potential interaction of *Aspergillus fumigatus* with airway immunity described above.

Bronchoscopic findings of IEPs can resemble those of other bronchial tumors depending on the tissue component [5], and small IEPs can be mistaken for malignancy or ABPA on chest CT. Therefore, pathological examination is required for a definitive diagnosis. However, diagnosing IEPs using bronchoscopic biopsy poses challenges, particularly with regard to the risk of bleeding. In this case, the eligibility of the polyp for biopsy was assessed through evaluations, including blood flow assessment using contrast-enhanced CT and endobronchial ultrasound and gentle pressure with biopsy forceps, and preparations were made to manage potential bleeding.

IEPs can be treated using various approaches, including conservative treatment, pharmacotherapy, bronchoscopic forceps extraction, Nd-YAG laser cauterization, and surgery [1]. Cryobiopsy, which has become widespread in recent years, is a potential option. However, the standard treatment has not been established, and precision medicine is often employed. Therefore, the appropriate treatment should be selected considering comorbidities. Conservative treatment has led to spontaneous resolution in 8% of cases [1]. Inhaled beclomethasone is effective for treating bronchial asthma in some cases [6]. Our patient was on long-term inhaled corticosteroids; however, the IEP did not resolve spontaneously for several months. Considering the preservation of lung function, bronchoscopic polypectomy was successfully performed, resulting in improvement. However, bronchoscopic intervention has been unsuccessful in some cases. Bjork et al. reported that transbronchoscopic intervention is difficult in case of large polyps with significant fibrous components, and surgery is recommended [3,9]. In this case, histopathological examination revealed that the pedunculated polyp had few fibrous components and measured approximately 1 cm in length, which likely facilitated its resection [3]. Cryobiopsy is also an option for tumor biopsies. However, for the effective management of the patient's bronchial asthma and ABPA, we prioritized EBFB as the treatment of choice to minimize invasion of the bronchial mucosa. A report indicating that EBFB was associated with a lower incidence of bleeding than cryobiopsy further supported the selection of EBFB [10].

Precise data on the recurrence of IEPs are scarce owning to the rarity of this disease. Eppinga et al. reported two cases with no recurrence after Nd-YAG laser removal of IEPs, but no follow-up period or methods were described [11]. However, follow-up reports of IEPs as a complication after bronchial thermoplasty and endobronchial ultrasound-guided transbronchial needle aspiration showed that the IEPs regressed after 5 weeks and 3 months, respectively [5,12]. In one case, complete disappearance occurred after 7 months, suggesting a low recurrence rate when the cause of polyp formation can be removed [5]. On the contrary, cases where the underlying factors contributing to polyp development cannot be resolved may be at a high risk of recurrence [13]. Although there is no evidence regarding follow-up intervals, given these reports, bronchoscopy or chest CT follow-up at intervals of approximately 3 months is considered appropriate within the first year of disease onset. In cases such as the present case, in which chronic lung disease is a complication, pulmonary function tests are required. Thereafter, follow-up based on symptoms and imaging findings may be advisable. The content and timing of these tests to effectively monitor for recurrence are important considerations for future research and clinical practice. This case is valuable because it is the first case in which symptoms, imaging findings, and changes in lung function during IEP management have been documented.

## 4 Conclusions

In patients with IEPs with chronic respiratory diseases such as ABPA, safely resolving the bronchial obstruction and maintaining lung function are essential for a better prognosis. Further studies are required to confirm the efficacy and safety for IEP management.

## Funding source

This research did not receive any specific grant from funding agencies in the public, commercial, or not-for-profit sectors.

## Data availability statement

The datasets generated and/or analyzed during the current study are available from the corresponding author on reasonable request.

## Patient consent

Consent forms were obtained.

## CRediT authorship contribution statement

**Fumihiro Kashizaki:** Conceptualization, Data curation, Formal analysis, Investigation, Project administration, Visualization, Writing – original draft, Writing – review & editing. **Kenji Konishi:** Data curation, Supervision. **Chihiro Yamada:** Data curation. **Shunsuke Okazaki:** Data curation. **Hao Chen:** Data curation, Visualization. **Atsushi Miyasaka:** Data curation. **Nanami Tsuchiya:** Data curation. **Akitomo Kikuchi:** Data curation. **Kentaro Yumoto:** Data curation. **Yui Kojima:** Data curation. **Hiroyuki Osawa:** Data curation, Supervision. **Harumi Koizumi:** Data curation, Supervision. **Kenichi Takahashi:** Supervision. **Takeshi Kaneko:** Supervision.

## Declaration of competing interest

The authors declare that they have no known competing financial interests or personal relationships that could have appeared to influence the work reported in this paper.
